# Social inequalities in the prevalence of self-reported chronic non-communicable diseases in Brazil: national health survey 2013

**DOI:** 10.1186/s12939-016-0427-4

**Published:** 2016-11-17

**Authors:** Deborah Carvalho Malta, Regina Tomie Ivata Bernal, Maria de Fatima Marinho de Souza, Celia Landman Szwarcwald, Margareth Guimarães Lima, Marilisa Berti de Azevedo Barros

**Affiliations:** 1 Nursing School, Universidade Federal de Minas Gerais (UFMG), Avenida Alfredo Balena, 190 Santa Efigenia, CEP – 30.130.100 Belo Horizonte, Minas Gerais Brazil; 2 Center for Epidemiological Research on Nutrition and Health School of Public Health, University of São Paulo, São Paulo, SP Brazil; 3 Department of Non-communicable diseases and Health Promotion - Ministry of Health of Brazil, Brasilia, Brazil; 4 Center for Scientific and Technological Information, Department of Health Information, Oswaldo Cruz Foundation, Manguinhos, Brazil; 5 Department of Public Health, School of Medical Sciences, UNICAMP, São Paulo, Brazil

**Keywords:** Chronic disease, Functional limitations, Inequalities, Health insurance, Low schooling level

## Abstract

**Background:**

Considering the high socioeconomic inequalities in Brazil related to occurrence of morbidity and premature mortality, the objective of this study was to analyze inequalities in self-reported prevalence of Non-Communicable Diseases (NCD) and in the physical limitations caused by these diseases, among the Brazilian adult population, according to sociodemographic variables.

**Methods:**

This was a population-based cross-sectional study that analyzed information on 60,202 individuals who formed a representative sample of Brazilian adults interviewed for the National Health Survey 2013. Disparities by schooling levels and possession of private health insurance were assessed by calculating the prevalence (P) and prevalence ratio (PR) of each of the 13 NCDs and any associated limitations, while controlling for other socioeconomic and demographic variables.

**Results:**

45 % of the Brazilian adult population reported having at least one NCD. The prevalence ratio was greater among women (1.24 CI 1.21-1.28), individuals over 55 years of age, individuals with low schooling levels (illiterate and incomplete elementary education) (1.08 CI 1.02-1.14) and people living in the Southeast (1.10 CI 1.04-1.16), South (1.26 CI 1.19-1.34) and Central-West (1.11 CI 1.05-1.18) regions of the country. Diseases such as diabetes (1.42 CI 1.13-1.47), hypertension (1.17 CI 1.06-1.28), stroke (2.52 CI 1.74-3.66), arthritis (1.4 CI 1.11-1.77), spinal problems (1.39 CI .1.25-1.56), and chronic renal failure (1.65 CI 1.10.2.46), were more prevalent among adults with low education. For most NCDs, greater reports of limitations were associated with lower schooling levels and lack of private health insurance.

**Conclusion:**

Populations with lower schooling levels and lack of private health insurance present higher prevalence of various NCD and greater degrees of limitation due to these diseases. Results reveal the extent of social inequalities that persist with regard to occurrence and the impact of NCDs in Brazil.

## Background

Non-Communicable Diseases (NCD), or chronic diseases, are one of the main health problems worldwide. They are responsible for 38 million deaths per year, which represent around 68 % of deaths worldwide [1, 2]. These diseases are associated with loss of quality of life, high degrees of functional limitations and reduced capacity to perform activities of daily living, and have important economic impacts on families, communities and society. Around 80 % of deaths occur in low or middle-income countries, and 29 % occur among individuals under the age of 60, thus accentuating health inequalities [1–3]. Over the last decade, NCDs have gained growing political recognition and priority on the agendas of international organizations and governments, and targets for their reduction were included in the Sustainable Development Goals (SDG) [1].

The increased burden of NCDs reflects rapid population aging coupled with negative effects of rapid urbanization, sedentary living and diets with high calorie content, along with marketing of tobacco and alcohol [4]. This burden especially affects the poorer and more vulnerable segments of the population. NCDs have greater effects on low-income populations because these groups are more exposed to risk factors and have less access to healthcare services and health promotion and disease prevention practices [1, 3]. Furthermore, a vicious circle is often created, in which family expenditures on NCDs is expanded, thereby reducing the availability of resources for necessities such as food, housing and education, among others. This may lead to a greater state of poverty for these families [2, 3, 5], which consequently worsens social inequalities [2, 3].

NCDs account for 72 % of deaths in Brazil [6]. The growth of NCDs in the past decades resulted from a rapid demographic transition -- with a large increase in the proportion of elderly people --, the nutritional transition associated with growth in obesity rates, and the exposure to various risk factors such as unhealthy diets, physical inactivity, tobacco and alcohol use [1, 2, 6]. In addition, studies have shown that social determinants such as education, occupation, income, gender, and ethnicity are associated with the prevalence of NCDs and their risk factors, aggravating the disease burden in vulnerable populations [6–9]. In this sense, *it* is important to measure these differences to support public policies that seek to reduce health inequalities [3, 5, 7, 8, 10].

With the aim of monitoring NCDs, the Brazilian Ministry of Health conducted the National Health Survey (NHS) in 2013, in partnership with the Brazilian Institute for Geography and Statistics (IBGE). This survey included information on NCDs, risk factors, access to health services, social determinants and others [11]. The NHS makes it possible to analyze inequalities in the distribution of NCDs in the country.

The objective of this study was to analyze inequalities in the self-reported prevalence of NCDs and the functional limitations caused by these diseases, among the Brazilian adult population, according to sociodemographic variables.

## Methods

Data from the NHS, a cross-sectional survey developed by the IBGE in partnership with the Ministry of Health, was analyzed. This comprised the most complete survey on health and its determinants ever conducted in Brazil [11, 12]. This household-based survey formed part of the IBGE’s Integrated System of Household-based Surveys (SIPD) and used the master sample of this system. This had greater geographic spread and consequently had greater precision of estimates, compared with the National Household Sampling Survey (PNAD) [11, 12]. The NHS was specifically designed to gather information on multiple aspects of health.

The sample design for the NHS was organized as clusters in three selection stages. In the first stage, the primary sampling units were selected by means of simple random sampling. These sampling units were formed by census tracts or sets of census tracts (when these census tracts contained few households). In the second stage, a fixed number of households ranging from 10 – 14 was selected in each primary sampling unit, again by means of simple random sampling. In each household sampled, one resident aged 18 years or over was selected by simple random sample to form the third selection stage.

The sample size was calculated to be approximately 80,000 households, and information on 62,986 households was gathered. In the calculation, the mean values, variances and sample design effects were taken into consideration, making the assumption of a non-response rate of 20 %.

Sampling weights for households and their residents were calculated as the product of the weight of the corresponding primary sampling unit and the inverse of the probability of selection of the household within the primary sampling unit. Weights were adjusted to correct for non-response and to calibrate the estimates in relation to total populations known from other sources. The weight of the selected resident was calculated as the product of the weight of the household and the number of eligible residents in the household (equivalent to the inverse of the selection probability) [12].

Data were gathered with the aid of handheld computers (personal digital assistants) that were programmed to verify inputted values. The NHS questionnaire was divided into three parts: information on the household; information on all the people living there (residents), provided by one of them (proxy); information on the resident selected, provided only by this person, who was an adult aged 18 years or over [11, 12].

The randomly selected adult resident answered an individual questionnaire that consisted of the following parts: resident’s perception of his own health; accidents and violence; lifestyles; diagnoses of NCD; women’s health; oral health; and medical attendance. In total, 60,202 interviews with adults selected within households were conducted.

In the present study, information from the selected resident in relation to the following was analyzed: NCD, i.e. arterial hypertension, diabetes, stroke, asthma and rheumatism; work-related musculoskeletal disorders; cancer; chronic renal failure, chronic spinal problems; depression. The question that was asked referred to previous medical diagnoses: a) “Has any doctor ever given you a diagnosis or arterial hypertension?” and successively for the other diseases, with the exception of: b) spinal pain, for which the question was “Do you have any chronic spinal problem?”; and c) depression, for which the question was “Has any doctor or mental health professional (such as a psychiatrist or psychologist) ever given you a diagnosis of depression?”.

The following estimates were calculated: prevalence of reporting at least one chronic disease, according to sociodemographic variables (age, sex, schooling level, color/race, geographic Region of residence, residence in an urban or rural region, and presence of private health insurance); the prevalence and prevalence ratio (PR) of each NCD among individuals aged 18 years or over, according to schooling level and private health insurance; and occurrences of severe or very severe degrees of limitation caused by the chronic disease, according to schooling level and private health insurance status. A comorbidity score was created, including those who reported two or three or more NCDs. This variable was stratified by level of education and health insurance.

The analyses included estimates of prevalence rates and the respective 95 % confidence intervals (CI). PRs were adjusted for sex, age and region. Poisson models were used to estimate PRs.

Sample design features were incorporated into the analysis program and the statistical software Stata 11.0 was used for data processing methods.

The microdata are open-access and available at: http://www.ibge.gov.br/home/estatistica/populacao/pns/2013_vol3/default_microdados.shtm.

The NHS project was approved by the National Research Ethics Commission CONEP), under the number 328.159, on June 26, 2013. All participants were given explanations about the survey, were asked whether they would be willing to participate, and gave their consent.

## Results

Based on the sample of 60,202 individuals, survey results revealed that 45.1 % of the Brazilian population aged 18 years and over reported having at least one chronic disease. The prevalence and prevalence ratios were higher among women (PR = 1.24 CI 1.21-1.28) and increased progressively with advancing age. In relation to the regions where the participants lived, higher morbidity was observed in the South (PR = 1.26 CI 1.19-1.34), Southeast (PR = 1.10 CI 1.04-1.16) and Center-West (PR = 1.11 CI 1.05-1.18) than in the North of Brazil (Table 1).

**Table 1:** Self-reported prevalence, prevalence ratio (PR) and 95 % confidence intervals for having at least one chronic non-communicable disease (NCD), according to sociodemographic conditions. Brazilian National Health Survey, 2013

Variables	*n*	%	Prevalence	PR_crude_	95 % IC	PR_adjusted_	95 % IC
Total	60,202		45.12				
Gender							
Male	25920	40.95	39.23	1.00		1.00	
Female	34282	59.05	50.37	1.28	(1.24,1.32)	**1.24**	**(1.21,1.28)**
Age							
18 to 24	7823	12.99	19.97	1.00			
25 to 34	13923	23.13	26.86	1.35	(1.22,1.48)	**1.34**	**(1.22,1.48)**
35 to 44	12817	21.29	41.09	2.06	(1.88,2.26)	**2.04**	**(1.86,2.24)**
45 to 54	10246	17.02	55.19	2.76	(2.53,3.02)	**2.73**	**(2.50,2.98)**
55 to 64	7681	12.76	67.7	3.39	(3.10, 3.71)	**3.34**	**(3.05,3.66)**
65 and over	7712	12.81	77.07	3.86	(3.54,4.21)	**3.79**	**(3.47,4.14)**
Race/Color							
White	24106	40.04	47.44	1.00		1.00	
Black	5631	9.35	43.87	0.93	(0.88,0.99)	0.99	(0.94,1.05)
Asian	533	0.89	38.59	0.85	(0.71,1.03)	**0.83**	**(0.71,0.98)**
Mixed	29512	49.02	42.89	0.91	(0.88,0.94)	**1.03**	**(1.00,1.07)**
Indigenous	417	0.69	48.66	1.11	(0.91,1.36)	**1.18**	**(1.00,1.39)**
Schooling level							
Higher education completed	7755	12.88	42.66	1.00		1.00	
High school education completed/higher education incomplete	19149	31.81	35.75	0.84	(0.79,0.89)	0.99	(0.93,1.05)
Elementary education completed/high school education incomplete	9215	15.31	39.68	0.93	(0.87,1.00)	1.05	(0.99,1.12)
Illiterate/elementary education incomplete	24083	40.00	56	1.31	(1.24,1.39)	**1.08**	**(1.02,1.14)**
Location of home							
Urban	49245	81.80	45.39	1.00		1.00	
Rural	10957	18.20	43.45	0.96	(0.92,1.00)	0.96	(0.92,1.01)
Region of residence							
North	12536	20.82	37.2	1.00		1.00	
Northeast	18305	30.41	42.22	1.14	(1.07,1.21)	1.05	(0.99,1.11)
Southeast	14294	23.74	46.06	1.24	(1.17,1.31)	**1.1**	**(1.04,1.16)**
South	7548	12.54	52.14	1.4	(1.31,1.50)	**1.26**	**(1.19,1.34)**
Center-West	7519	12.49	43.95	1.18	(1.11, 1.26)	**1.11**	**(1.05,1.18)**
Private health insurance							
No	47073	78.19	43.95	1.00		1.00	
Yes	13129	21.81	48.73	1.11	(1.07,1.15)	**1.01**	**(0.98,1.05)**

PR adjusted for sex, age and region

Results graphed in bold correspond to a statistically significant prevalences

The correlation of each chronic disease with schooling levels is shown in Table 2. Higher percentages of diabetes (1.42 CI 1.13-1.47), hypertension (1.17 CI 1.06-1.28), stroke (2.52 CI 1.74-3.66), arthritis (1.4 CI 1.11-1.77), spinal problems (1.39 CI .1.25-1.56), and chronic renal failure (1.65 CI 1.10.2.46), were observed among individuals whose schooling levels were lower as compared with those with completed higher education. While the prevalence of stroke was higher in all the lower strata of schooling, the prevalence of musculoskeletal disorders and cancer was higher among those with higher schooling levels.

**Table 2:** Prevalence (P), prevalence ratio (PR) and 95 % confidence interval (95 % CI) of chronic diseases or conditions among individuals aged 18 years or over, according to schooling level. Brazilian National Health Survey, 2013

Morbidities	Measure	Illiterate/elementary education incomplete	Elementary education completed/high school education incomplete	High school education completed/higher education incomplete	Higher education complete
Arterial hypertension	P	31.14	16.66	13.36	18.16
	PR(^a^)	**1.17**	1.11	0.99	1.00
	95 % CI	**(1.06-1.28)**	(0.99-1.24)	(0.89-1.10)	
Diabetes	P	9.61	5.36	3.44	4.18
	PR(^a^)	**1.42**	**1.59**	1.19	1.00
	95 % CI	**(1.13-1.77)**	**(1.23-2.06)**	(0.93-1.51)	
Stroke	P	2.74	0.82	0.79	0.58
	PR(^a^)	**2.52**	**1.71**	**2.01**	1.00
	95 % CI	**(1.74-3.66)**	**(1.09-2.67)**	**(1.29-3.14)**	
Arthritis	P	9.26	5.50	4.12	4.74
	PR(^a^)	1.22	**1.40**	1.18	1.00
	95 % CI	(0.99-1.50)	**(1.11-1.77)**	(0.94-1.47)	
Asthma	P	4.07	4.42	4.57	4.96
	PR(^a^)	0.90	0.93	0.94	1.00
	95 % CI	(0.74-1.11)	(0.72-1.20)	(0.76-1.16)	
Spinal problems	P	24.58	15.79	13.94	14.73
	PR(^a^)	**1.39**	**1.18**	1.08	1.00
	95 % CI	**(1.25-1.56)**	**(1.04-1.34)**	(0.97-1.22)	
Musculoskeletal disorders	P	1.99	1.91	2.68	3.83
	PR(^a^)	**0.59**	**0.57**	0.77	1.00
	95 % CI	**(0.44-0.79)**	**(0.41-0.79)**	(0.58-1.02)	
Depression	P	8.61	6.95	6.40	8.71
	PR(^a^)	1.02	0.94	0.86	1.00
	95 % CI	(0.86-1.20)	(0.78-1.13)	(0.73-1.01)	
Cancer	P	2.31	1.11	1.16	3.00
	PR(^a^)	**0.46**	**0.46**	**0.60**	1.00
	95 % CI	**(0.35-0.61)**	**(0.32-0.67)**	**(0.43-0.82)**	
Chronic kidney failure	P	2.08	1.16	0.95	0.97
	PR(^a^)	**1.65**	1.38	1.22	1.00
	95 % CI	**(1.10.2.46)**	(0.89-2.12)	(0.80-1.87)	
2 NCDs	%	15,20	9,78	7,87	11,68
	RP(^a^)	1,00	0,98	0,84	1,00
	IC(95 %)	(0.87,1.16)	(0.83,1.16)	**(0.73,0.98)**	
3 and more NCDs	%	12,95	6,31	4,85	6,47
	RP(^a^)	1,34	1,21	1,05	1,00
	IC(95 %)	**(1.13,1.59)**	(0.98,1.49)	(0.86,1.29)	

(^a^)PR adjusted for sex, age and region

Results graphed in bold correspond to a statistically significant prevalences

Having three or more NCDs was most frequent among the population with the lowest education levels (1:34 CI 1.13,1.59).

From analysis of the degree of limitation resulting from these diseases by schooling levels, for all the diseases studied, with the exception of chronic renal failure, the prevalence of functional limitations increased with decreasing schooling levels. Limitations caused by arterial hypertension, diabetes, asthma, musculoskeletal disorders were more than five times greater among those with lower versus higher schooling. On the other hand, limitations caused by stroke, arthritis and depression were simply more prevalent among those with lower schooling (Table 3).

**Table 3:** Prevalence (P), prevalence ratio (PR) and 95 % confidence interval (95 % CI) of severe and very severe degrees of limitation among individuals aged 18 years or over, according to schooling level. Brazilian National Health Survey, 2013

Morbidities	Measure	Total	Illiterate/elementary education incomplete	Elementary education completed/high school education incomplete	High school education completed/higher education incomplete	Higher education complete
Arterial hypertension	P	4.71	6.06	4.48	3.25	0.65
	PR(^a^)		**8.94**	**6.78**	**4.92**	1.00
	95 % CI		**(4.48-17.82)**	**(3.04-15.-11)**	**(2.29-10.58)**	
Diabetes	P	7.04	8.18	9.04	4.40	1.42
	PR(^a^)		**5.70**	**6.33**	**3.04**	1.00
	95 % CI		**(2.27-14.34)**	**(2.27-17.65)**	**(1.02-9.08)**	
Stroke	P	25.48	29.92	17.94	15.11	10.67
	PR(^a^)		**2.63**	1.71	1.45	1.00
	95 % CI		**(1.22-5.69)**	(0.64-4.57)	(0.54-3.86)	
Arthritis	P	17.10	20.48	14.98	13.19	8.68
	PR(^a^)		**2.34**	1.73	1.51	1.00
	95 % CI		**(1.24-4.40)**	(0.86-3.49)	(0.75-3.02)	
Asthma	P	6.00	8.18	5.32	6.15	0.94
	PR(^a^)		**7.66**	**5.95**	**7.00**	1.00
	95 % CI		**(2.89-20.34)**	**(1.71-20.68)**	**(2.41-20.31)**	
Spinal problems	P	16.42	21.12	15.51	10.91	7.01
	PR(^a^)		**2.76**	**2.27**	**1.63**	1.00
	95 % CI		**(2.00-3.83)**	**(1.55-3.33)**	**(1.14-2.33)**	
Musculoskeletal disorders	P	15.73	27.98	14.82	11.46	4.48
	PR(^a^)		**5.85**	**3.22**	**2.54**	1.00
	95 % CI		**(2.85-12.00)**	**(1.38-7.49)**	**(1.24-5.18)**	
Depression	P	11.83	16.11	9.77	8.91	6.45
	PR(^a^)		**2.59**	1.46	1.31	1.00
	95 % CI		**(1.58-4.25)**	(0.79-2.69)	(0.78-2.20)	
Cancer	P	10.32	10.23	14.74	13.80	5.08
	PR(^a^)		2.20	3.05	**2.69**	1.00
	95 % CI		(0.94-5.14)	**(1.02-9.12)**	**(1.07-6.72)**	
Chronic kidney failure	P	11.85	13.17	**4.68**	9.70	19.09
	PR(^a^)		0.75	**0.22**	0.43	1.00
	95 % CI		(0.35-1.64)	**(0.07-0.71)**	(0.14-1.29)	

(^a^)PR adjusted for sex, age and region

Results graphed in bold correspond to a statistically significant prevalences

Table 4 shows the prevalence and prevalence ratio of chronic diseases according to the presence of private health insurance. Stroke was more prevalent among individuals without private health insurance (PR = 1.30 CI 1.00-1.69), while musculoskeletal disorders and cancer were more prevalent among those with private insurance. Having private health insurance was not associated with increased comorbidity.

**Table 4:** Prevalence (P), prevalence ratio (PR) and 95 % confidence interval (95 % CI) of NCD among individuals aged 18 years or over, according to whether they had private health insurance. National Health Survey, 2013

		Private health insurance	
		No	Yes
Arterial hypertension	P	20.76	23.38
	PR(^a^)	1.05	1.00
	95 % CI	(0.99-1.09)	
Diabetes	P	5.93	7.19
	PR(^a^)	1.01	1.00
	95 % CI	(0.89-1.14)	
Stroke	P	1.57	1.39
	PR(^a^)	**1.30**	1.00
	95 % CI	**(1.00-1.69)**	
Arthritis	P	6.14	7.26
	PR(^a^)	0.97	1.00
	95 % CI	(0.85-1.09)	
Asthma	P	4.22	4.96
	PR(^a^)	0.91	1.00
	95 % CI	(0.78-1.05)	
Spinal problems	P	18.40	18.68
	PR(^a^)	1.04	1.00
	95 % CI	(0.96-1.13)	
Musculoskeletal disorders	P	2.08	3.54
	PR(^a^)	**0.69**	1.00
	95 % CI	**(0.56-0.84)**	
Depression	P	7.19	9.03
	PR(^a^)	0.95	1.00
	95 % CI	(0.84-1.07)	
Cancer	P	1.54	2.72
	PR(^a^)	**0.75**	1.00
	95 % CI	**(0.61-0.93)**	
Chronic kidney failure	P	1.33	1.71
	PR(^a^)	0.88	1.00
	95 % CI	(0.67-1.16)	
2 NCDs	%	11,00	13,05
	RP(^a^)	0,96	1,00
	IC(95 %)	(0.88,1.05)	
3+ NCDs	%	7,97	9,89
	RP(^a^)	1,01	1,00
	IC(95 %)	(0.91,1.13)	

(^a^)PR adjusted for sex, age and region

Results graphed in bold correspond to a statistically significant prevalences

Table 5 shows the presence of severe and very severe degrees of limitation caused by morbidities, according to private health insurance status. The prevalence of limitations was higher among those with no private health insurance, for individuals with chronic renal failure (PR = 3.42 CI 1.27-9.22), asthma (PR = 2.94 CI 1.25-6.88), cancer (PR = 2.59 CI 1.41-4.76), hypertension (PR = 1.90 CI 1.22-2.94)), spinal problems (PR = 1.51 CI 1.25-6.88) and depression (PR = 1.48 CI 1.05-2.07).

**Table 5:** Prevalence (P), prevalence ratio (PR) and 95 % confidence interval (95 % CI) of severe and very severe degrees of limitation among individuals with NCDs, according to whether they had private health insurance. Brazilian National Health Survey, 2013

Morbidities	Measurements	Total	Private health insurance	
			Yes	No
Arterial hypertension	P	4.71	2.84	5.39
	PR(^a^)		1.00	**1.90**
	95 % CI			**(1.22-2.94)**
Diabetes	P	7.04	8.00	6.66
	PR(^a^)		1.00	0.80
	95 % CI			(0.47-1.35)
Stroke	P	25.48	24.37	25.79
	PR(^a^)		1.00	1.11
	95 % CI			(0.67-1.85)
Arthritis	P	17.1	14.56	18.08
	PR(^a^)		1.00	1.23
	95 % CI			(0.92-1.66)
Asthma	P	6.00	2.54	7.32
	PR(^a^)		1.00	**2.94**
	95 % CI			**(1.25-6.88)**
Spinal problems	P	16.42	12.19	17.81
	PR(^a^)		1.00	**1.51**
	95 % CI			**(1.25-1.83)**
Musculoskeletal disorders	P	15.73	14.39	15.73
	PR(^a^)		1.00	1.14
	95 % CI			(0.75-1.74)
Depression	P	11.83	8.551	13.17
	PR(^a^)		1.00	**1.48**
	95 % CI			**(1.05-2.07)**
Cancer	P	10.32	5.128	13.29
	PR(^a^)		1.00	**2.59**
	95 % CI			**(1.41-4.76)**
Chronic kidney failure	P	11.85	4.023	15.14
	PR(^a^)		1.00	**3.42**
	95 % CI			**(1.27-9.22)**

(^a^)PR adjusted for sex, age and region

Results graphed in bold correspond to a statistically significant prevalences

## Discussion

The present study based on data from the 2013 NHS showed that 45 % of the Brazilian adult population reported having at least one NCD, and that the most frequent chronic diseases were hypertension, spinal/back pain, diabetes, arthritis/rheumatism, depression, and bronchitis/asthma. The presence of at least one NCD was more frequent among women, individuals 55 years and older, individuals with low schooling levels (illiterate and incomplete elementary school) and people living in the southeastern, southern and central-western regions of Brazil. Five of the diseases surveyed were more prevalent in the stratum of lower schooling. Physical limitations caused by NCDs were reported more frequently among those with lower schooling and those without any private health insurance. These results indicate the presence of social inequalities in the distribution of NCDs in the Brazilian population, and greater physical limitation due to these diseases among more vulnerable populations.

Morbidity data are important for managing healthcare systems and for planning and evaluating healthcare service provision. Analysis of such data with a focus on inequalities may indicate ways to address existing disparities [2, 3]. However, such information is often unavailable in many middle- and lower-income countries [1].

The literature from high-income countries suggests higher prevalence of NCDs in less educated populations, a finding that is consistent with the results found here for Brazil [4, 11].

A survey conducted in eight countries (Denmark, France, Germany, Italy, Japan, Netherlands, Norway and the United States), investigating NCD morbidity (including hypertension, diabetes, ischemic heart disease, allergies, arthritis, congestive heart failure and chronic pulmonary disease), found that 55.1 % of the adults aged 18 and over reported at least one chronic condition [13]. The prevalence of having at least one NCD observed in the present study (45 %) was higher than that observed in previous studies based on the 2003 National Household Sampling Survey (PNAD) [14] (40 %) and the 2008 PNAD [7] (40.6 %). This higher level is likely the result not only of continued aging of the population but also from expansion of access to diagnoses of these diseases in Brazil over the past decade.

The greater prevalence of the majority of the self-reported NCD found among women was concordant with the literature [13, 15, 16]. This situation has been attributed to the fact that women seek out and use healthcare services more than men do, thus resulting in greater opportunity for being diagnosed. Studies have attributed this to women’s greater perception of the physical signs and symptoms of these diseases, which is facilitated by attending healthcare clinics more frequently [7, 16, 17]. The difference in prevalence between the sexes was 20 % in 2008 (adjusted PR = 1.20) [7] and increased slightly to 24 % in 2013 (adjusted PR = 1.24). These differences may be explained by higher life expectancy among women, resulting in an increased disease burden, as well as an increased demand for health services and thus a greater opportunity for diagnosis among women [14, 16, 17].

The greater occurrence of NCD with increasing age is coherent with the literature and results from the aging of the population and the greater disease burden among elderly people [1, 6, 7].

Analyses in Brazil from the PNAD 2008 revealed that, after adjustment for age, sex and other variables, there was higher prevalence of reporting at least one NCD among people living in urban rather than rural areas, and among those living in the southern region of the country [7]. This finding was attributed to greater access to healthcare services in these areas. Comparison with previous PNAD surveys shows that there has been a progressive increase in the prevalence of NCD among the rural population: from 37.7 % in 2003 [14] to 39.6 % in 2008 [7] and 43.4 % in 2013. This trend is indicative expansion of access to medical diagnoses for people living in rural areas.

Findings from this study also indicate higher NCD prevalence among people with low versus higher levels of education. This pattern was detected in several studies conducted in developed countries [18–20]. Also in Brazil, previous studies have shown similar results including those from the World Health Survey 2003 [21] and PNAD 2003 [7]. In 2008, NCDs were more prevalent among individuals with lower education levels, except for tendonitis/tenosynovitis and cancer, which occurred predominantly among people with higher education levels [7, 14].

In India, a recent survey showed the opposite; groups with higher income had higher self-reported NCD prevalence as compared with low-income groups, probably due to under-diagnosis and underreporting of disease among the poor [22], since populations with higher socioeconomic status in low and middle income countries usually have better access to health care [23, 24]. There may be organizational, social, cultural and/or financial barriers that limit access to health services among populations of low socioeconomic status, all of which could affect the opportunity to diagnose NCDs [18, 19, 25, 26].

Epidemiological studies on self-reported non-communicable diseases may therefore underestimate the NCD prevalence in groups of low socioeconomic levels. It is therefore recommended that correction measures be used [22]. Unlike the findings from India and some other middle income countries [22, 25], the NHS 2013 showed greater prevalence of self-reported NCDs in the population of low schooling levels in Brazil. Thus, Brazil’s situation is more similar to what is seen in populations in high income countries like the United States, Canada and European countries [18–20]. This result is likely explained by greater access to healthcare services in Brazil for poorer populations, due to the National Health System (SUS), which is public, universal and free-of-charge. The SUS includes broad segments of the population and has been associated with reductions in socioeconomic inequalities in health and healthcare [27, 28].

NHS 2013 revealed higher prevalence of hypertension, diabetes, spinal problems, arthritis, chronic renal failure and stroke among those with lower schooling levels. These associations had also been observed in PNAD 2008 [7], with the exception of stroke, which was not investigated. On the other hand, higher prevalence of cancer and musculoskeletal disorders were observed in the strata with greater schooling, as had been observed in 2008 [7].

In addition, higher prevalence of smoking, obesity, poor nutrition and low levels of physical activity in the population with less education explains the higher prevalence of hypertension, diabetes and chronic renal failure among these populations [2, 6, 29, 30].

It is recognized that pains and musculoskeletal problems affect a large portion of the population resulting in economic impact and loss of quality of life. Brazilian studies [31] showed that diseases of the spine/back affect a large portion of the population with less schooling. A review study indicated that educational level has an impact on the duration and recurrence of episodes of back pain. Individuals with more education have more favorable evolution of back pain [32].

Only two diseases presented greater prevalence in those with higher schooling: cancer and musculoskeletal disorders. In relation to cancer, a study conducted in European countries [19] showed a profile similar to that found in the present study, with lower cancer prevalence among individuals with low schooling levels. In Brazil, studies have shown that musculoskeletal disorders and tendonitis are more frequent among people of higher socioeconomic levels [7, 33, 34], which may be connected with their greater presence in the labor market, greater risk of developing the disease, greater awareness of the risks of repetitive exertion, and greater access to diagnoses and longer life expectancy.

NHS 2013 revealed that for all the diseases investigated with the exception of chronic renal failure, significantly greater prevalence of severe or very severe limitations were observed among individuals with lower schooling levels.

In relation to musculoskeletal diseases, the literature suggests that their greater impact in more socially vulnerable populations contributes towards worsening these individuals’ disabilities and exacerbating difficulties finding and remaining in work [33]. It has been recognized that a high percentage of the population demands healthcare services because of these problems and that the frequency of limitations on daily activities is high, including time off work, retirement due to disability and days spent bedridden [35].

Even in relation to cancer and musculoskeletal disorders, which are more prevalent in the strata with greater schooling, the impact in terms of severe and very severe limitations is greater in the segment with lower schooling. Social inequalities relating to such limitations have many causes: lower and later access to services, attendance of lower quality, fewer resources, poor living conditions, lack of information for enabling good treatment, lack of follow-up and poor disease management. It has been suggested that appropriate restructuring of attendance and care for patients could reduce inequalities relating to limitations of daily activities [36]. Studies evaluating inequalities in the limitations caused by NCDs remain scarce, especially in less developed countries [36].

The choice to adjust rates by age, sex and region was important and justified by the rapid demographic transition in the country, with different gains in life expectancy by sex. Women live longer, due to deaths by external causes among men, making it important for the adjustment of NCD prevalence by sex. There are also significant regional differences in the age composition. Adjustment by Region also becomes relevant considering regional differences in access to health services as well as differences in the educational attainment.

There was higher prevalence of some NCDs (cancer and tendonitis) in the population without private health insurance [7]. Previous studies have identified that people with health insurance have more access to health care, lower prevalence of risk factors for NCDs and greater access to preventive cancer screening [37–39].

In recent years, the portion of Brazilians in the formal labor market increased and this increased access to employer-paid private health insurance [37, 38]. The expansion of coverage of public and private health services could explain the reduction of differences in NCD prevalence among individuals with and without private health insurance. However, when considering the degree of limitation, individuals with NCDs who do not have health insurance presented a higher prevalence of intense physical limitation for several NCD (hypertension, asthma, spinal column problems, depression, cancer, chronic renal failure) than those who have insurance. This finding may be the result of delayed access to health services or lack of resources for the treatment and management of these diseases by people without private health insurance [37–39].

This study has advantages and limitations. The main advantage is the large nationally representative sample of the population. Another advantage of the study is its internal validity, given that the NHS produced good quality data [11, 12].

On the other hand, the study has limitations inherent to its cross-sectional nature. The results are based on self-reported data and thus subject to recall bias. In addition, disparities in disease prevalence are associated with differential access to health services among groups of different socioeconomic levels [22]. Thus, a higher prevalence may be related to opportunities for diagnoses made by health professionals, which could partially explain some of the regional and socioeconomic differences observed here [36, 40].

Moreover, our analysis did not include an important indicator of SES, income, since this information was not available when our analysis was implemented. Thus, the magnitude of differences according to schooling level observed in the present study may have been underestimated, given that the segments with lower schooling level tend to have less access to healthcare services and to diagnostic tests and hence underreport the presence of morbidities.

There may also have been differences in the prevalence found in relation to those of other studies because of differences in the number and type of health problems and chronic diseases that were included in the survey; the age group investigated; the sampling method; the questions and words used in the questionnaire; the access to healthcare services among the population surveyed; and the type of respondent (the person concerned or another member of the family speaking on his behalf), among others.

In 2011, Brazil launched a Strategic Action Plan for NCDs, establishing actions and targets to reduce premature mortality (deaths between the ages of 30 and 69) by 2 % per year, and reduce the prevalence of associated risk factors [8, 9]. Premature mortality from NCDs has been declining in Brazil [6, 9, 29]. Several public policies encouraging healthy diet, reducing salt in food, creating public spaces to support physical activity, and mandating smoke free environments, in addition to investments in primary care and diagnostic and specialty services have been implemented [6, 8, 9].

## Conclusion

This study found that substantial social inequalities relating to the prevalence of NCDs and revealed that these inequalities severely affect the impact that these diseases have on people’s lives. The prevalence of severe or very severe limitations resulting from these NCDs were up to five to eight times greater in the segment with lower versus higher schooling.

These results indicate that to reduce inequalities, there is a need not only to expand access to diagnosis and treatment within the socially more vulnerable segments of society but also to improve healthcare quality and promote healthier behaviors. Thus, expansion of the public healthcare system needs to focus increasingly on prevention and control of chronic diseases, with special attention to the impact of morbidities on individuals’ daily activities.

Studies on social inequalities relating to NCD are important for monitoring inequalities in prevalence and for alerting and directing healthcare services towards providing special attendance for segments of the population with higher prevalence of diseases and which suffer more greatly through the impacts of NCDs.

## Abbreviations

CI: Confidence interval
CONEP: National Research Ethics Commission
IBGE: Brazilian Institute of Geography and Statistics
NCD: Non communicable disease
PNAD: National Household Sampling Survey
PNS: National Health Survey
PR: Prevalence ratio
SDG: Sustainable Development Goals
SES: Socioeconomic status
WHO: Word Health Organization
